# Supplementary figures and images for: Transcriptional differentiation of Trypanosoma brucei during in vitro acquisition of resistance to acoziborole
Source: PLoS Negl Trop Dis. 2021 Nov 9;15(11):e0009939. doi: 10.1371/journal.pntd.0009939 (PMC8648117; doi:10.1371/journal.pntd.0009939)

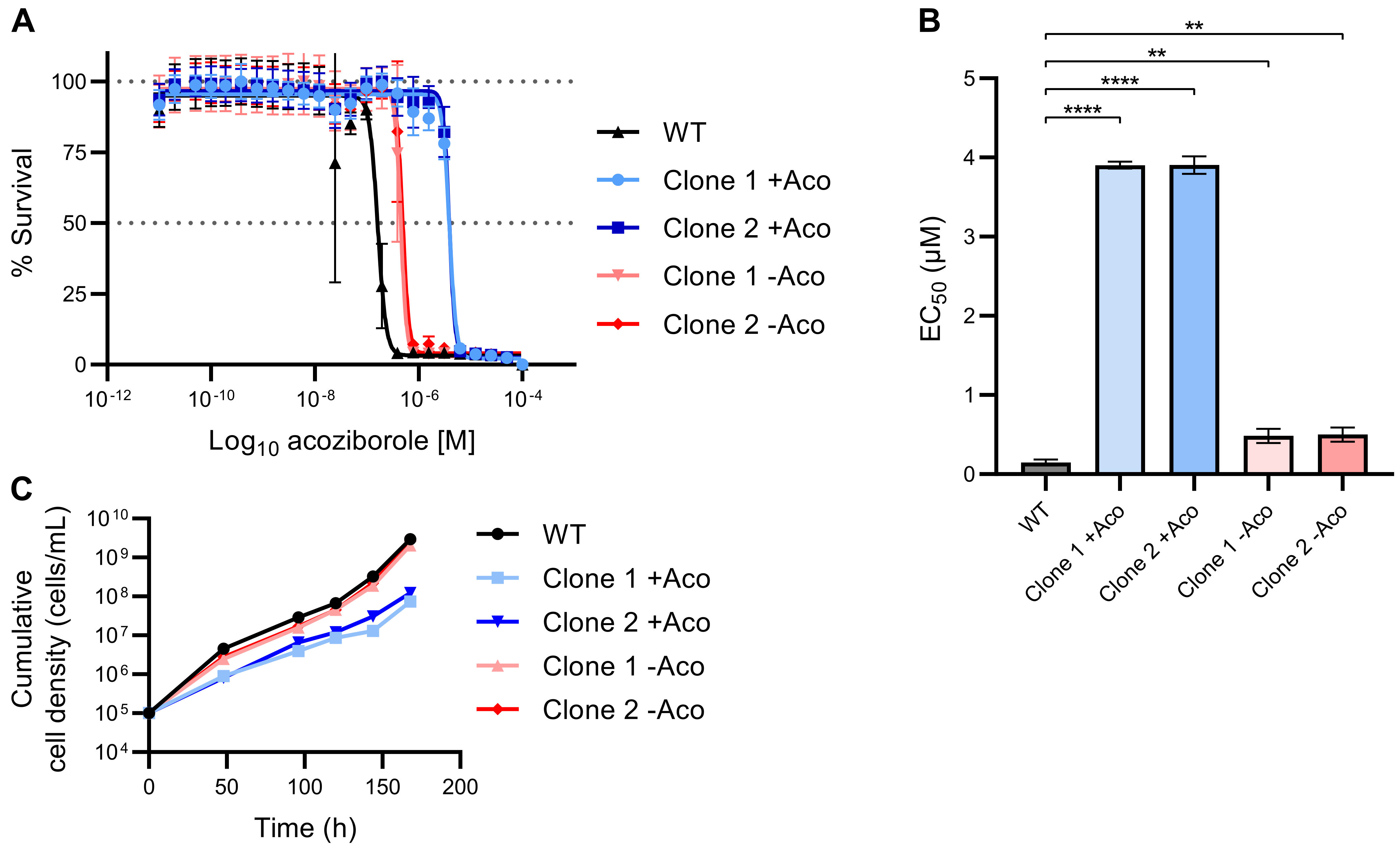

Supplement: S1 Fig — Two clones of the AcoR line were grown for 14 days in the presence (+Aco) or absence (-Aco) of 4.96 μM acoziborole, prior to testing sensitivity to the benzoxaborole, compared to a wild-type (WT) control. A) Sigmoidal dose-response curves of two clones in the presence or absence of acoziborole with a wild-type control. A shift to the right indicates increased acoziborole resistance. B) Mean EC50s from three independent experiments. Acoziborole resistance is reversed in AcoR cells grown without drug pressure, although resistance is still significant compared to that of wild-type cells (Student’s T-test, **p < 0.01, ****p < 0.0001). C) Cumulative growth curves of wild-type T. brucei and AcoR cells in the presence or absence of 4.96 μM acoziborole. Mean doubling times were 7.6 h, 10.8 h, 12.7 h, 7.1 h and 6.8 h for WT, clone 1 +Aco, clone 2 +Aco, clone 1 -Aco and clone 2 -Aco, respectively. (TIFF) [file pntd.0009939.s001.tiff]

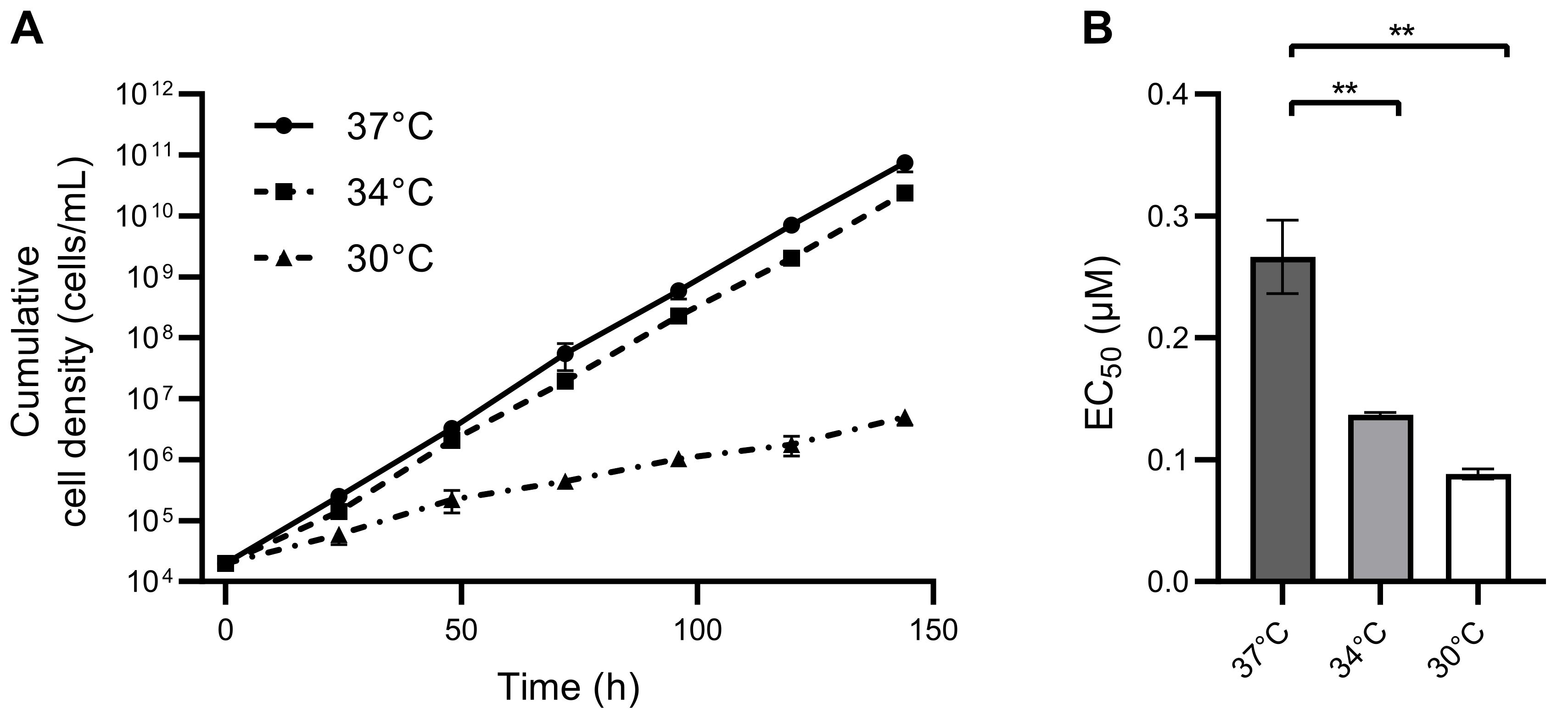

Supplement: S2 Fig — Wild-type T. brucei was grown at lower temperature (34°C and 30°C) to test the effect of reduced growth rate on acoziborole sensitivity. A) Growth was significantly reduced at 30°C (doubling times: 7.0 h, 6.8 h and 18.5 h at 37°C, 34°C and 30°C, respectively, calculated using an Malthusian growth model) only. B) Acoziborole sensitivity was significantly increased in T. brucei when cultured under conditions of lower temperature and reduced growth rate. Statistics performed by unpaired t-test, **p < 0.01. (TIFF) [file pntd.0009939.s002.tiff]

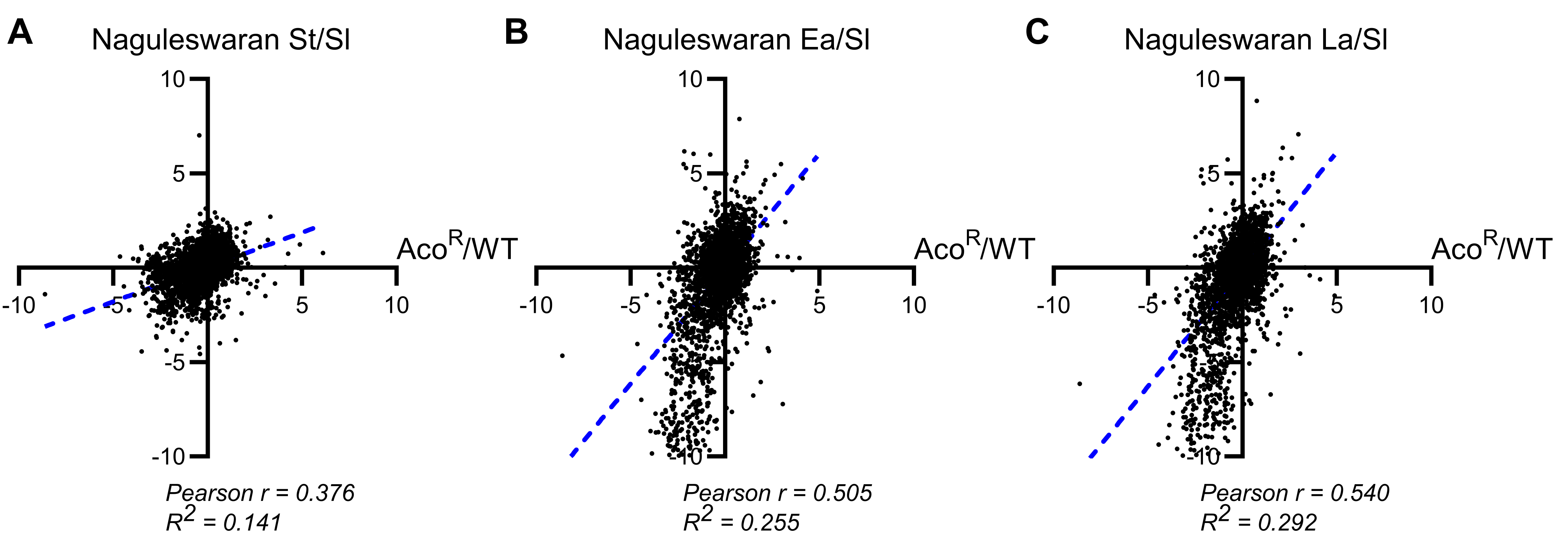

Supplement: S3 Fig — Transcriptomics data generated by Naguleswaran and colleagues [48] was processed by the same means as the data generated in this study and log2 fold changes (as calculated by DESeq2) of AcoR vs. WT were compared to log2 fold changes of stumpy vs. slender (A), early procyclic vs. slender (B) and late procyclic vs. slender (C). In this study, early procyclics showed coordinated social motility whilst late procyclics did not [48]. Significance of correlations between the datasets were tested by linear regression (R2; blue dotted line) and Pearson correlation (Pearson’s r). Abbreviations: St: stumpy; Sl: slender; Ea: early procyclic; La: late procyclic. (TIFF) [file pntd.0009939.s003.tiff]
