# Supplementary material for: Transcriptional differentiation of Trypanosoma brucei during in vitro acquisition of resistance to acoziborole
Source: PLoS Negl Trop Dis. 2021 Nov 9;15(11):e0009939. doi: 10.1371/journal.pntd.0009939 (PMC8648117; doi:10.1371/journal.pntd.0009939)
Supplement: Attachment — Submitted filename: acoziborole_resistance_manuscript_reviewers_comments_FINAL.pdf [file pntd.0009939.s008.pdf]

University of Glasgow | College of Medical, Veterinary & Life Sciences

Dear Prof. Phillips and PLoS Neglected Tropical Diseases editorial team,

Thank you for your reviews of our manuscript "Transcriptional differentiation of Trypanosoma brucei during in vitro acquisition of resistance to acoziborole". We are pleased with the response to our work.

Although the reviewers seemed to find the work of interest, there were some major issues raised, in particular with the transcriptomics analysis and resistance phenotype we described. The comments were constructive, for which we are grateful. In this revised manuscript, we present a reanalysis of the transcriptomics data, in addition to several key experiments regarding the reversibility of the acoziborole-resistance phenotype. Importantly, our findings are unchanged, but we do feel, as the reviewers have suggested, that the additional data provide greater insight. We have addressed each individual point raised by all three reviewers and in addressing these, we have expanded the manuscript by i) revising the transcriptomics analysis and statistics (including a quantitative comparison with publicly available stumpy- and procyclic-form data, and revision of figure 2, table 1, table 2 and S1 table, as well as insertion of a new supplementary figure), ii) insertion of a new supplementary figure outlining the reversibility of the resistance phenotype, iii) insertion of a new supplementary figure outlining drug sensitivity in T. brucei cultured at reduced growth rate via means of temperature reduction, iv) discussion of limitations, especially with regards to lack of protein level analysis, and v) clarification of text as suggested by reviewer comments.

Thank you for considering our manuscript, we hope the revised manuscript is suitable for publication in PLoS Neglected Tropical Diseases.

Kind regards,

Pieter Steketee

Michael Barrett

Professor Michael P Barrett
Institute of Infection, Immunity & Inflammation
120 University Place, Glasgow, G12 8TA, UK
Email: Michael.Barrett@glasgow.ac.uk
Tel : +44(0)141 330 6904
Fax : +44(0)141 330 4600
http://www.gla.ac.uk/researchinstitutes/iii/

The University of Glasgow, charity number SC004401

Reviewer's Responses to Questions

Key Review Criteria Required for Acceptance

Reviewer #1: The methods are appropriate but more extensive analysis is needed for the transcriptome and genome, using the well-annotated TREU927 genome as a basis.

> *We thank the reviewer for their constructive comments and have provided answers in the relevant sections below.*

Reviewer #2: Yes to all questions asked except for the question about ethical/regulatory concerns where the answer is no. In the summary and general comments my concerns are raised - most can be addressed by the authors without further experiments but there are straightforward immunoblotting or classic TEM analysis for the authors to consider and which would lift the work.

> *We have provided answers to Reviewer #2 in the 'Summary and General Comments' section.*

Reviewer #3: The objective of the study was to examine possible mechanism of acoziborole resistance in Trypanosoma brucei through the in vitro generation of resistant lines. The study design is appropriate and the trypanosome strain chosen is the one that is most often used for these kinds of in vitro analyses. There is one issue with the trypanosome strain used and that is that the Lister 427 line is monomorphic and has some defects in the normal trypanosome differentiation pathway, and since the mechanism of resistance uncovered in this study appears to involve a differentiation-type switch in gene expression it would have been useful to use a pleiomorphic line as well. However the existence of this mechanism couldn't have been predicted at the start of the study and so the strain used was appropriate given the more likely mechanisms of resistance (these being drug efflux or overexpression of the protein target). It may be the monomorphic nature of the strain used that ensured there was no morphological change to accompany the transcriptomic changes.

> *We thank the reviewer for their comments. We agree that in hindsight, using a pleiomorphic T. brucei line would have provided further insight into the resistance phenotype uncovered in this study. We have inserted an extra paragraph into the discussion to highlight the valid points made by the reviewer (lines 120–122 and 393–400).*

Results

Reviewer #1: Benzoxaboroles are likely to be decisive tools in the future control of African trypanosome diseases. The primary target of the drugs that are currently in late-stage trials is the cleavage and polyadenylation factor CPSF3, but various aspects of the drug effects remain unexplained- notably, changes in methylated metabolites. Resistance is always a potential problem for chemotherapy, but for the human candidate, acoziborole, no lines with more than low-level resistance have been described. In this paper, the authors describe T. brucei that are roughly 20-fold resistant to acoziborole. They show changes in methylated metabolites, with no further effects upon treatment. Although the mechanism is-as before, completely unknown, the results do hint at some polypharmacology which may contribute to the severe difficulty in obtaining resistance. The resistant cells also grow very slowly, which would almost certainly preclude selection in the field. However the slow growth could also be the reason why the cells tolerate the drug.

The authors claim that the cells are procyclic-like in their transcriptomes but actually they don't know because they have not done a quantitative comparison with procyclic- or stumpy-form transcriptomes. From the growth kinetics, morphology and transcriptome results, I suspect that these cells are actually showing some characteristics of stumpy-form trypanosomes, more than procyclics.

Major modifications required: The sequencing alignment was done with the most recent genome of the cognate strain, Lister427. While this is essential for examining VSG expression sites, it is pretty useless for everything else. This is because the annotation of that genome is really poor. It is impossible to tell, from these Tables, what gene products are actually affected. More importantly, it is not possible compare the transcriptome results presented here with any of those previously published. It is therefore essential to supply equivalent tables made using the TREU927 genome annotation, which is way superior for everything except expression sites. From my own experience, I can say that it will be essential to re-do the sequence alignments, because the Tables of homologues that can be downloaded from TritrypDB are incomplete. One table (or sheet) should include raw read counts so that it is possible for others to do quantitative comparisons without repeating the alignments. Please include both the open reading frames and the 3'-UTRs; this will enable genes with similar ORFs, but different regulation (such as PGKB and PGKC) to be distinguished. The authors must also then do a comparison with published stumpy-form and procyclic-form transcriptomes, to see which most closely resembles the resistant cells; and also look at cell-cycle regulation. It is indeed revealing that in the discussion, the authors revert to the 927 numbers - referring the reader to supplementary Tables on which these numbers cannot be found! In fact in Table S1 the authors do not even show the 427 gene IDs of the increased and decreased transcripts. Exactly the same applies to the SNP and indel analysis. You can't tell if something is important if you don't know the gene function. The whole analysis also needs to be done again using 927. You can check afterwards whether the changes are simply differences between 427 and 927.

We agree with the reviewer that the TREU 927 in terms of annotation, is the gold standard for the T. brucei genome. We initially aligned to the recent (Siegel) assembly of the Lister 427 T. brucei genome, as this was the relevant assembly for the in vitro strain we were using in this study. However, we have taken the reviewer's comments into account and developed a new approach which combined the TREU 927 assembly core chromosomes (v50.0) as well as the Lister 427 BES contigs (not the 2018 Siegel version; also v50.0), which contain the relevant VSG expression sites for the strain used in this study.

We carried out the alignment with the same methodology as previously outlined, using this hybrid genome as reference. Reads were counted HTSeq-count with the "ID = exon" parameter, which takes into account CDS as well as UTRs. In addition, we also aligned data from two recent studies (PMIDs: 30307943 and 29606092). The former contained reads from both slender (ascending parasitaemia) and stumpy (peak parasitaemia) form parasites from in vivo infection (with the caveat that the strain used was EATRO 1125 AnTat1.1). The latter contained reads from slender, stumpy and procyclic form parasites derived from culture (with the same caveat). All data was analysed using DESeq2 and fold changes of Aco^R/WT (this study) and Stumpy/Slender (Silvester et al., study) were compared by linear regression (R^2 = 0.284) and Pearson correlation (r = 0.533). In addition, fold changes of Aco^R/WT were also compared to fold changes of Stumpy/Slender (R^2 = 0.141; r = 0.376), early-PCF/Slender (R^2 = 0.255; r = 0.505) and late-PCF/Slender (R^2 = 0.292; r = 0.540) from the Naguleswaran et al. dataset. We have included a new panel in Figure 2 with the Silvester et al. data, as well as a new supplementary figure (S3 Fig) with the comparisons to the Naguleswaran et al. data.

Given the direct relevance of the Siegel PacPio assembly to the strain used in this study, we chose to keep the SNP and Indel analysis as previously submitted. However, we have included relevant TREU927 orthologues and their product descriptions in the supplementary file.

We note that the overall conclusions from the transcriptomics analysis have not been altered by re-analysis of the data.

Table 1: Please compress this to include only one example of the repeated genes. Simplify the annotations (not just copy-paste of the database entry) and show GeneDB numbers for the homologues (if present) in TREU927. The RHS proteins are known to have various different functions: which ones are affected here? Which nucleoside transporter is it, most are now characterised? For example, Tb427_000257300.1 turns out to most likely be an ESAG3 pseudogene, but I had to blast the nucleotide sequence onto 927 to find out. What are all the other "hypothetical proteins" in the Table? Aligning to 927 may well tell you. The results would also appear to suggest a decrease in expression site transcription. Please show a screen shot of the read alignment across the active expression site, then it will be possible to tell.

We have simplified and compressed both Table 1 and Table 2, using the revised transcriptome dataset

Tables in text: Put all Padj values in the same format. Tables everywhere: These results are not accurate to 9 significant figures, please remove superfluous decimal places, it makes the Tables much smaller and much easier to read.

We agree on the preferred formatting suggestion. We have made the suggested amendments including removal of superfluous decimal places and consistency in the Padj values and we hope this satisfies the reviewer's requests.

Reviewer #2: Yes, but some revision of Fig 1 is needed and revision of Table 2 highly encouraged - see 'Summary and General Comments'

We have provided answers to Reviewer #2 in the 'Summary and General Comments' section

Reviewer #3: Most of the results are clearly presented, there are a couple of minor issues: Lines 424-426 Fig 1. Legend to part B does not refer to the figure but talks about changes in EC50 whereas the figure is about doubling times. Also the doubling times should be given quantitatively as numbers rather than just showing growth curves (Lines 122-123).

We thank the reviewer for raising this, issues with the legend for figure 1 were raised by all three reviewers, which we now deal with. We have revised the figure legend for Fig 1 to now reference all three panels. In addition, we have calculated doubling times and provided these in the Fig 1 legend, as well as in the main text.

Line 426 The C is missing where the legend should refer to part C.

Thanks to the reviewer for spotting this. We have amended the legend for Figure 1 to include references to panels A, B and C.

Fig 5A - surely the authors could have got a better image of a single cell rather than one that is folded back on itself.

We have revised this figure and inserted a new image from the same experiment. We hope this is more acceptable to the reviewer.

Conclusions

Reviewer #1: It seems very likely that the cells are acoziborole resistant because they are growing very slowly. Slow-growing parasites have a lower requirement for RNA processing than normal cells. This could also explain the resistance to sinefungin. If this is true then the question is, WHY are these cells growing slowly? Some hints may come from the reanalysis of the transcriptome and genome proposed above.

The reviewer makes a good point, regarding slow growth reducing the requirements of RNA processing. To test whether slow growth alone could lead to acoziborole resistance, we carried out further experiments with T. brucei Lister 427 cells incubated at decreased temperatures, and we have generated a new supplementary figure to summarise these data.

Whilst we first attempted growth at 25°C, cell growth was almost completely abolished, and it was noted that cells exhibited severe difficulties with cell division. We therefore attempted further work at different temperatures – 34°C and 30°C, compared to 37°C. As shown in S2 Fig, whilst growth rate is not significantly altered at 34°C (doubling times of 7.0 h and 6.8 h for 37°C and 34°C, respectively), cells grew slowly at 30°C (doubling time of 18.5 h), and drug sensitivity assays were carried out at both temperatures. Notably, reductions in incubation temperature appear to correlate with increased acoziborole-sensitivity, indicating that slow growth (and the cellular stresses associated with reduction in temperature) is not enough to generate acoziborole resistance, at least when that slow growth is induced by a temperature shift. Indeed, cells incubated at lower temperatures hypersensitize. We note, though, that lower temperature (in the case of 34°C) also led to hypersensitisation when growth rate wasn't significantly reduced, so can't rule out temperature and growth rate having separate effects that we cannot tease out in these experiments.

We have added further sentences to the results and discussion sections of the manuscript to describe and discuss the above and hope that this satisfies the reviewer's comments.

The conclusion that there is a "master-switch" controlling differentiation is not supported by the data and should deleted. Instead, I suspect that the changes seen in these cells may be related to a prolonged stress response, as suggested in a recent publication (10.1016/j.pt.2020.11.003). The cited publication also includes numerous references to stresses that can cause stumpy-like changes. Please read, consider, and cite. The question would then be why the cells retain this phenotype. Perhaps the resistant cells have mutated so as to make the stressed state permanent? could this explain the increased susceptibility to some other drugs? Is the phenotype lost really rapidly when selection is removed, or do the authors have to wait to select revertants? If the latter, the change must be genetic and the authors might be able to find it from the genotype results - especially if they also sequence revertants.

We have removed the sentence referring to a "master-switch" controlling differentiation. We thank the reviewer for mentioning the publication on prolonged stress responses and have taken this into consideration, as well as the studies cited by the review. We have added a new paragraph to the discussion section of the manuscript (lines 381–392).

Reviewer #2: Overall yes, but clarity in some areas is called for in 'Summary and General Comments'.
Public health relevance is addressed nicely.

We thank the reviewer for their comment.

Reviewer #3: The major problem with the conclusions is that all of the gene expression data is based on RNA levels and it is known that trypanosomes employ translational control as well so increased transcript numbers may not result automatically in increased protein levels. It would have

been useful if the authors had demonstrated that some of the procyclic specific genes were actually expressed at the protein level (for example the procyclins/PARP/EP/GPEET) as there are antibodies available and it would be easy to demonstrate surface expression of these proteins. Or if they had employed ribosomal profiling rather than just RNA-seq to demonstrate that the upregulated PCF genes were being translated. In this context one experiment that should be done is to check the protein expression level of CPSF3 since they dismiss the idea of overexpression by saying there were no changes in RNA level but if the CPSF3 mRNA is more efficiently translated in a "PCF-like" context then they may be missing the actual resistance mechanism.

Unfortunately, we do not have any protein-related work. We agree on its desirability, but did not carry out protein related work when the study was being performed. We do consider it unlikely that the parasites manifest a proteome that is distinct from that suggested by transcriptomic changes, but lack evidence, hence we add a comment, that "in the lack of verification of the levels of proteins, the presence of which is inferred from the transcriptional data, these findings remain tentative.

Due to the inaccessibility of the mass spectrometer, coupled to the fact that the first author no longer works in Glasgow, and general problems associated with the covid19 pandemic, we were not able to perform proteomics work within the time frame set by the journal as the outer limit for resubmission.

We have included a new sentence in the discussion section to address this comment, which reads as follows (lines 342–343): "Unfortunately, in the absence of data other than transcript abundance we cannot definitively exclude a role for CPSF3."

With the metabolomics data they discuss several metabolites with changes in the resistant cell lines eg carnitine, orotate etc but they do not mention whether these metabolic changes link to any upregulated or downregulated genes in the RNA-seq data. This should be done and it should be made clear if there are any significant changes in pyrimidine metabolic genes for example or genes related to L-carnitine metabolism or pathways L-carnitine is involved in.

We have amended the results section to compare the transcriptomics and metabolomics data in more detail. Whilst carnitine levels are elevated in resistant cells, we found no significant changes in transcripts associated with genes involved in carnitine metabolism (e.g. carnitine O-acetyltransferase) we therefore assume that these elevated levels correspond to increased uptake (unfortunately we have found no studies identifying carnitine transporters) and are indicative of increased mitochondrial metabolism.

With regards to pyrimidine metabolism, there were very few changes at the transcriptome level apart from several significantly reduced genes (nucleoside diphosphatase, Tb927.7.1930, Log_2 fold change = -1.80; uridine phosphorylase, Tb927.8.4430, -1.47; nucleoside hydrolase, Tb11.v5.0270, -1.02). However, we could not ascertain whether these transcript changes could be responsible for the orotate/dihydroorotate observation in the metabolomics data, and therefore they were not discussed in detail.

We have added the observations described above to the results section (lines 283–297).

I cannot see anywhere where the authors have tested the stability of this resistance phenotype - is the phenotype lost in the absence of acoziborole, and if so how rapidly does it revert? In this light it would also have been interesting to see whether any live trypanosomes could have been recovered from an infected mouse (Lines 290-291).

We agree with both reviewers 1 and 3 that testing the stability is important. As mentioned above, performing additional experiments has created logistical hurdles. However, we have now shown that when left without drug pressure, the resistance phenotype significantly reverts, and growth rate returns relatively quickly (lines 141–148), hence we consider the phenotype to be reversible. Although it is possible, therefore, that putting the parasites under the pressure of an intact immune system might also have stimulated selection of the

revertants, we did not perform any animal experiments, having judged it unlikely that the parasites would survive in animals whilst manifesting the transcriptional profile we observed (where VSG expression is down-regulated and procyclin expression is up-regulated, leading to the assumption the parasites would be destroyed by the immune system and thus not ethically justifiable to test in mice).

Editorial and Data Presentation Modifications

Reviewer #1: Minor corrections (grammar or unclear):

"There were small but significant decreases observed in three copies of the CBP1 gene". This should be changed to "There were small but significant decreases observed in mRNA encoding CBP1" followed by an reminder of what the function of CBP1 is, with an appropriate reference.

This has been amended

"HAT case number has" -> "HAT case numbers have" OR "The HAT case number has"

This has been amended to "HAT case numbers have"

for stage 1 and stage 2 treatment of HAT -> for treatment of stage 1 and stage 2 HAT

This has been amended

downregulated, -> down-regulated; upregulated -> up-regulated.

We have amended 2 instances of "downregulated" to "down-regulated" and three instances of "upregulated" to "up-regulated"

"T. brucei brucei is infectious to livestock" -> T. brucei brucei and T. brucei rhodesiense are infectious..

This has been amended

the disease can be fatal.. -> the disease is usually fatal.. (I think this is still true)

This has been amended

procyclic (PCF) insect vector stages in the tsetse fly -> delete "insect vector"

This has been amended

"reduction in case number of the disease caused by T. b. gambiense" -> "reduction in T. b. gambiense case numbers".

This has been amended

"Finally, abundance of PAD proteins" - should be "the abundances of transcripts encoding PAD proteins..." - plural needed, and proteins were not measured.

This has been amended

Line 271: "|the cpsf3 gene" Gene should be in capital letters and italics.

This has been amended

"in clinical practise as.." -> "practice" ("Practise" is a verb.)

This has been amended

Reviewer #2: My view is that operationally this is a major 'minor revision'.

Reviewer #3: There are some minor changes required:

Line 70: Only eflornithine is biologically species specific, it is policy that dictates the use of the other drugs, for example suramin is not used because of coinfection with filarial nematodes, not because T. gambiense is resistant to it. Also, the word disease should be inserted in front of stage-specific to distinguish it from parasite stages.

> *This has been amended, the sentence now reads as follows: "Furthermore, some of those that are used currently are species (eflornithine) and disease stage-specific, with notable issues in administration routes, toxicity and emerging resistance [8-12]."*

Line 117: Lister 427 is a monomorphic strain with a defective differentiation pattern - authors should comment on this in the discussion, in particular as regards mitochondrial activation for example.

> *We agree with the reviewer that this is an important point to make, especially given the results of this study. We have therefore expanded the first paragraph of the results section to include a comment regarding the monomorphic nature of the Lister 427 strain. This addition reads as follows (lines 120–122): "Notably, this strain is known to be monomorphic, and is incapable of differentiation through normal routes to procyclics via stumpy forms – including a failure to trigger mitochondrial oxidative phosphorylation…"*

Line 123 numbers should be given for doubling times.

> *We have amended references to parasite growth to include doubling times*

Line 267 "step chance" should be "step change"

> *This has been amended*

Line 284 insert the word partial before differentiation as there was no morphological differentiation.

> *This has been amended*

Lines 424-426 Fig 1. Legend to part B does not refer to the figure but talks about changes in EC50 whereas the figure is about doubling times.

> *Thanks for pointing this out. We have re-written the legend for Fig 1. As mentioned above, we have rewritten the legend for figure 1 which now references all three panels in the figure.*

Summary and General Comments

Reviewer #1: See above for overall comments. The study is interesting but without more detailed analysis of the transcriptomes and genotype, including effects of removing the selection, the results are difficult to interpret. If the cells are resistant simply because they are growing more slowly, the reasons for the slow growth need to be examined.

Reviewer #2: The authors describe the generation and characterization of an acoziborole-resistant bloodstream Trypanosoma brucei cell line. They report co-resistance to sinefungin and hypersensitivity to other drugs. Overall, it's a straightforward manuscript, but one that I enjoyed reading and the work will be of value and interest to others in the community. However, as listed below, there are areas that need tidying before the work is suitable for publication.

Line 123, cell doubling times lengthen (i.e. increase) not reduced (Fig 1B). In the legend to Fig 1B, the growth curve is not described – rather B) in the legend is referring to Fig 1C. And finally with

respect to Fig 1B the cell densities per ml seem rather high (and don't tally with what's in the Materials and Methods for in vitro culture. Or am I looking at an in vivo growth curve? If in vitro are the resistant clones grown up in the presence of drug or not? None of the cultures, if these are batch cultures, appear to have reached stationary phase – this is particularly of note for the resistant cultures since at what density do they plateau out?

We apologise for the oversight regarding the legend for Figure 1B, we have amended and added information about the growth curve. With regards to the high density values, these growth curves are cumulative. Cells were passaged to 2 × 10^4 cells/mL when density reached ~2 × 10^6 cells/mL and dilution factor was taken into account when calculating cell density at the next time point. We have added in sentences to clarify this in both the figure 1 legend as well as the Materials & Methods sections. All Aco^R cells in figure 1B were cultured in the presence of acoziborole, apologies for our lack of clarity in this, we have added this information to the figure legend.

We previously did not grow resistant cells at densities higher than 2 × 10^6 cells/mL. However, in order to answer the reviewer's question regarding stationary phase, we carried out new experiments in order to test whether these resistant cells plateau out at a particular density. Acoziborole-resistant cells reached stationary phase at 2-3 ×10^6 cells/mL, similar to wild-type Lister 427 T. brucei. This is likely due to exhaustion of essential medium components such as glucose.

More generally, 4 clones from the same resistant population are analysed, so the wider question is how representative is the resistance mechanism described in the current work? Of course, were a second resistant population analysed and it presented a different resistance phenotype, one would be content with reading the description of two different resistance phenotypes – so I have no problem with the single resistant population analysed but I see no indication that expression, at the protein level, of the expressed VSG is reduced, but much appears made of the changes in VSG expression seen at the level of transcriptomics. Is the 221 VSG actually translated at a lower level in the resistant population versus the parent? Unless the authors are clearer on the effect of resistance on VSG expression, the reader is left hanging – I'm not sure for instance that if say endo/exocytic rate were reduced this wouldn't affect the amount of VSG on the actual cell surface.

We do not wish to propose this as "the" mechanism for resistance to acoziborole. Indeed, as acknowledged above, we feel that the likely phenotypic changes (albeit unproven as we acknowledge) of loss of the bloodstream form proteins including VSG would render these parasites unviable in immunocompetent mammals. It is this an intriguing observation we would like to bring to the attention of others without necessarily impacting directly on an understanding of acoziborole resistance as an issue in African trypanosomes. We believe the notion of a large scale transcriptional shift that brings with it resistance to an antimicrobial drug can also be of relevance in other systems, again a reason why we would like to publish the observations.

Did the authors complete any TEM – is the classic appearance of the VSG coat (e.g. as noted by Vickerman) evident all around the cell or is that surface coat now patchy or the cell surface changed in some other way evident in EM?

Unfortunately, TEM analysis was not carried out during this study. However, the point made by the reviewer is a good one, and we have added a sentence to the discussion section to mention that further work on the resistant cell population should include TEM analysis (lines 371–374).

Table 2 – would be helpful to know by reading (rather than dig through TriTrypDB) whether the several hypotheticals are T. brucei-specific trypanosomatid/kinetoplastid-specific, chromosome-internal or sub-telomeric. More details please.

Based on comments by reviewer 1, we have completely revised the transcriptomics data, which has now been aligned to a hybrid genome consisting of core chromosomes from TREU

927 ('gold standard' reference) and BES contigs from Lister 427. As a result, Table 2 has been revised and primarily shows TREU927 genes which have more accurate annotation.

At the level of proteomics or immunoblotting is there any evidence for translation of procyclic marker proteins e.g. PPDK, PARP, transporters in the resistant cell line – the authors talk about a 'differentiation event' (or phenotype) (line 284) but I don't see any evidence really for anything other than a bloodstream cell with an altered transcriptome and odd kinetoplast-nucleus distance and the latter perhaps is a function of slow cell growth.

How long did the authors growth the resistant mutants for – does the growth rate ever speed up? Is the resistance phenotype (or slow growth) retained after drug pressure is removed? These details were also missing but would be helpful to know.

We thank the reviewer for their comments. Resistant cells were grown in the presence of acoziborole for several months without a change in growth rate, which initially led us to start analysis of the cell line. All three reviewers commented on the reversibility of the resistance phenotype. We have therefore carried out further analysis to test whether resistance is lost after drug pressure is removed and these results are presented in a new supplementary figure (S1 Fig). We found that after 14 days, there is significant hypersensitisation to acoziborole accompanied by an increased growth rate. However, these cells are still ~3-fold more resistant to acoziborole than wild-type cells. We have discussed these results in the context of references provided by reviewer 1, in particular the similarities between stress response and differentiation pathways in trypanosomes.

Reviewer #3: This study provides an interesting insight into novel mechanism of drug resistance in African trypanosomes and also provides new information about the differentiation process. It is interesting that the procyclin genes are upregulated as well as other PCF specific genes, since the procyclins are transcribed from specialise loci by RNA polymerase I like the VSG genes so the transcriptional switch posited by the authors must affect the RNA Pol I stage regulated system as well as RNA Pol II transcribed genes. This resistance mechanism is unlikely to operate easily in the field as PCF-like forms are unlikely to survive in an immunocompetent mammalian host as the authors state.

There are two major weaknesses, one is that the authors have not demonstrated that any of the upregulation at the RNA level leads to upregulation of the encoded proteins, trypanosomes display translational control so it is not automatic that increased mRNA leads to increased protein levels. The authors should mention this caveat in their discussion. The second is that they have identified a phenomenon - "transcriptional differentiation" which accompanies the acquisition of acoziborole resistance but they have not identified a mechanism whereby this leads to resistance. Overall the study is well-performed within the above limitations and is both novel and significant in increasing our understanding of the plasticity of trypanosome phenotypes.

We thank the reviewer for this comments. We agree that there are limitations to this study which we hope can be addressed by other experts in the field, should it be of interest. We also agree that these limitations were not stated clearly in our submission, and have therefore added several sentences to the discussion section to highlight them.
