# Supplementary material for: Transcriptional differentiation of Trypanosoma brucei during in vitro acquisition of resistance to acoziborole
Source: PLoS Negl Trop Dis. 2021 Nov 9;15(11):e0009939. doi: 10.1371/journal.pntd.0009939 (PMC8648117; doi:10.1371/journal.pntd.0009939)
Supplement: Attachment — Submitted filename: acoR_manuscript_reviewers_comments_round2.pdf [file pntd.0009939.s009.pdf]

University of Glasgow | College of Medical, Veterinary & Life Sciences

Dear Prof. Phillips and PLoS Neglected Tropical Diseases editorial team,

Thank you for your reviews of our manuscript "Transcriptional differentiation of Trypanosoma brucei during in vitro acquisition of resistance to acoziborole". We are pleased that our revisions satisfied the reviewers, and delighted that all three have recommended publication.

However, Reviewer 1 had a few valid comments and considerations for the revised submission. To address these, we have make edits in the manuscript where requested (as outlined below), and in addition, based on comments regarding the transcriptome data comparisons, we have expanded S1 table to include these comparisons to aid the reader, and in the interest of open science.

Thank you for considering our manuscript, we hope the revised manuscript is suitable for publication in PLoS Neglected Tropical Diseases.

Kind regards,

Pieter Steketee

Michael Barrett

Professor Michael P Barrett
Institute of Infection, Immunity & Inflammation
120 University Place, Glasgow, G12 8TA, UK
Email: Michael.Barrett@glasgow.ac.uk
Tel : +44(0)141 330 6904
Fax : +44(0)141 330 4600
http://www.gla.ac.uk/researchinstitutes/iii/

The University of Glasgow, charity number SC004401

Reviewer's Responses to Questions

Summary and General Comments

Reviewer #1: This paper is very much improved. There are only a few minor things now, which have resulted from new introductions to the text.

I noted that the authors talk about procyclic and stumpy in the Introduction but don't actually say what stumpy forms are, which would be a problem for potential readers who don't work on trypanosomes but are interested in the benzoxaboroles. A couple of sentences that say what stumpy forms are and list genes important for (and markers for) stumpy differentiation are needed. Also, somewhere the authors need to describe the morphological differences between the three forms considered.

> *We agree with the reviewer's comments and have added a paragraph to the introduction section of the revised manuscript (lines 64–81). The paragraph reads as follows: "African trypanosomes display a complex life cycle that includes procyclic (PCF) stages in the tsetse fly as well as a mammalian-infective bloodstream (BSF) stage. Pleomorphic T. brucei strains are rapidly dividing in the BSF stage, with a long and slender morphology, but possess the ability to differentiate into non-dividing short stumpy forms, which are pre-adapted to survival in the insect vector [5]. This developmental transition is triggered by a quorum sensing mechanism involving a "stumpy induction factor", recently identified as oligopeptide uptake mediated by the TbGPR89 transporter [6]. Differentiation between the various life cycle stages is accompanied by morphological changes, as well as global shifts in transcriptome and metabolome (including downregulation of BSF-specific variant surface glycoproteins – VSGs – and associated expression sites) [5,7-12]. Characterisation of the T. brucei life cycle stages has yielded the identification of several stage-specific markers. For example, stumpy-form T. brucei exhibits upregulation of transcripts encoding PAD (protein associated with differentiation) [9], as well as downregulation of histones, DNA replication/repair and cytoskeleton-related transcripts, indicative of their non-proliferative state [7,13]. In addition, both stumpy forms and PCFs express transcripts encoding EP and GPEET procyclins in place of VSGs [14]. Differentiation to stumpy form and PCF also involves downregulation of transcripts associated with glycolysis, as the parasite begins to shift to mitochondrial metabolism [8,12]."*
>
> *Regarding morphology, we also agree that differences between the life cycle stages needs to be introduced. In order to not make the introduction too long, we have elected to give a brief outline in the relevant results section. This outline reads as follows (lines 335–338): "The morphology of T. brucei differs significantly depending on life cycle stage. BSFs are long and slender, whilst stumpy-forms are rounded and short, as their name implies. In contrast, PCFs are elongated and exhibit decreased distance between kinetoplast (mitochondrial DNA) and nucleus [51]."*

Line 116 – change to "a partial switch towards procyclic mRNA abundances". I find the word "phenotype" a bit too strong. Many readers might assume that "phenotype" means morphology, which was not much changed.

> *This has been amended, the sentence now reads as follows: "In summary, acoziborole resistance, in vitro, appears to be characterised by global transcriptomic changes, leading to a partial switch towards procyclic mRNA abundances in resistant cells."*

Line 1272 – “transcript” not “transcription”.

We believe the reviewer was referring to line 357 (line 367 in the revised manuscript). We agree with the change, and this has been amended.

“Transcripts associated with proteins involved in stage-specific regulation of mRNA (e.g. reduction in PARN-1; Tb927.8.2850) were also significantly altered. “ The evidence that PARN1 is involved in stage-specific regulation of mRNA is based on artificial over-expression. The effects on differentiation have not been measured because the only paper (Utter et al, not cited) is with non-differentiation-competent cells. A procyclic knock-out grew almost normally and RNAi had no effect in bloodstream forms. Actually, looking at the results of the microarrays, they are really weird -a whole cluster from one particular region of chromosome 9 is all up about 2-fold. It’s inducible so unlikely to be a gene duplication. The only genes affected that are linked to differentiation are the BARP genes, which are actually a marker for some salivary gland epimastigotes. in that context, there’s an increase in HAP2 mRNA in the acoziborole resistant cells, which suggests to me a stress response since it’s a gamete protein. No harm in mentioning PARN1 but I’d be careful about saying what it does. If you want to find proteins implicated in control of gene expression in the bloodstream-stumpy-procyclic transition you would be on more solid ground with the known markers and signalling proteins, and with ZC3H11 and ZC3H22 (Tb927.7.2680).

We thank the reviewer for bringing the information on PARN-1 to our attention. We have removed the reference to PARN-1 from the discussion section. In place of this reference, we have gone for more solid ground when referencing transcripts involved in stage-specific regulation of mRNA, specifically, the zinc finger proteins mentioned by the reviewer, as well as PAD1 and PAD2, as transcripts encoding both are increased in acoziborole-resistant cells.

The sentences related to the Reviewer’s comment now read as follows (lines 388–391): “Transcripts associated with proteins involved in stage-specific regulation of mRNA (e.g. increases in PAD1, PAD2, ZC3H11 and ZC3H22; Tb927.7.5930, Tb927.7.5940, Tb927.5.810 and Tb927.7.2680, respectively) were also significantly altered.”

I was intrigued by the set of proteins that are affected in the slender-vs-stumpy or slender -vs-procyclic comparisons, but not in the acoziborole-resistant cells. For stumpy forms they seem to be dominated by cytoskeletal proteins and histones - the stumpy cells aren’t dividing and the resistant cells are.

We thank the reviewer for bringing this to our attention, as we had not made this connection. We believe this would be of interest to mention, and have therefore added in a sentence to the results section to highlight this observation. The sentence reads as follows (lines 249–251): “Interestingly, a large portion of genes down-regulated in stumpy form cells, but not in acoziborole-resistant cells, were associated with cell division. For example, histones and cytoskeletal proteins (S1 Table).”

Furthermore, given that these data could hold further clues to the potential mechanisms underlying the acoziborole-resistance phenotype, we have added two worksheets to S1 Table, which contain the slender-vs-stumpy and slender-vs-procyclic comparisons to the Aco^R-vs-wild-type transcriptome. These worksheets contain the DESeq2 outputs as well as an extra column containing the “distance from X=Y” for each gene. In theory, if $\log_2$ fold changes between the datasets were equivalent, all genes would fall on an X=Y line on a scatter plot comparing the data. The values in this new column are the calculated deviation from the X=Y line. Positive values indicate that the $\log_2$ FC is higher in the Aco^R-vs-WT dataset and conversely, negative values indicate that the $\log_2$ FC is higher in the previously published datasets.
